# Motor function in Parkinson's disease and supranuclear palsy: simultaneous factor analysis of a clinical scale in several populations

**DOI:** 10.1186/1471-2288-6-26

**Published:** 2006-06-13

**Authors:** Pieter M Kroonenberg, Frans J Oort, Glenn T Stebbins, Sue E Leurgans, Esther Cubo, Christopher G Goetz

**Affiliations:** 1 Department of Education and Child Study, Wassenaarseweg 52, Leiden University, Leiden, The Netherlands; 2 Department of Medical Psychology, Academic Medical Centre, University of Amsterdam, The Netherlands; 3 Department of Neurological Sciences, Rush-Presbyterian-St. Luke's Medical Center, Chicago, Illinois, USA

## Abstract

**Background:**

In order to better understand the similarities and differences in the motor behaviour of different groups of patients, their scores on the Motor Examination section of the Unified Parkinson's Disease Rating Scale (UPDRS) were analysed simultaneously. The three groups consisted, respectively, of patients with Parkinson's disease (PD) on medication, patients with Parkinson's disease withdrawn from anti-parkinsonian medication for at least 12 hours, and patients diagnosed with a specific Parkinsonism syndrome: Progressive Supranuclear Palsy (PSP).

**Methods:**

A total of 669 consecutively sampled patients from three separate hospital-based clinics participated (294 PD on medication; 200 PD off medication: 175 PSP). The Motor Examination section of the UPDRS was administered by neurologists at the three participating clinics. The patient scores on each item were recorded. To assess similarities and differences among the components of the UPDRS in these samples, we performed simultaneous or multigroup factor analysis on the covariance matrices of the three groups. In addition, it was investigated whether a single model for the Motor Examination section of the UPDRS could be developed which would be valid for all three groups at the same time.

**Results:**

A single six-dimensional factor solution was found that fitted all groups, although this was not straightforward due to differences between the tremor-at-rest variables. The factors were identified as Tremor-at-rest, Postural Tremor, Axial Dysfunctioning, Rigidity, Left Bradykinesia and Right Bradykinesia. The analysis also pointed to a somewhat lower lateralization in bradykinesia for PSP patients. The groups differed in intensity of motor impairment, especially with respect to Tremor-at-Rest, but the overall relationships between the variables were shared by the three groups. In addition, apart from the common factor structure evidence of differences in body part-specific and motor-specific variances was found.

**Conclusion:**

From a clinical point of view, the analyses showed that using the Motor Examination section of the UPDRS is also appropriate for patients with PSP, because the correlational structure of the items is directly comparable to that of Parkinson's patients. Methodologically, the analysis of all groups together showed that it is possible to evaluate similarities and differences between factor structures in great detail.

## Background

Parkinson's Disease (PD) is one of the most common neuro-degenerative disorders, estimated to afflict approximately 1% of Americans over the age of 65 (Bennett, Beckett, Murray, Shannon, Goetz, Pilgrim, & Evans, 1996; Tanner, 1994) [1,2]. PD is associated with the loss of dopamine-producing cells in the substantia nigra pars compacta, resulting in low levels of dopamine in the striatum. Lack of dopamine in the striatum provokes the primary clinical manifestations of PD: tremor, rigidity, bradykinesia (the gradual loss of spontaneous movement; slowness of voluntary movements), and postural reflex impairment (Hornykiewicz, 1966; Bernheimer, Birkmayer, & Hornykiewicz, 1973) [3,4].

### Motor examination of the unified Parkinson's disease rating scale

The Unified Parkinson's Disease Rating Scale (UPDRS) is a validated quantification of Parkinson's Disease signs and symptoms. (Fahn & Elton, 1987) [5], which assesses six primary areas of disability associated with PD. The UPDRS is the most widely used measurement tool for motor impairment in PD. The Motor Examination section of the UPDRS is the primary focus of this paper, because it is only this section that provides a detailed assessment of PD motor impairment based on physical examination of the patient. The main motivation behind the present study was to assess the ability of the scale to reliably measure movement disorders across multiple types of diseases. In addition, the aim was to develop a model for motor behaviour describing the structure of the items of the Motor Examination section which would be valid for all samples. The basis for this exercise was that the scale's domain is motor function assessment, so it should provide similar factor structures regardless of the specific illness. The clinical import of a positive finding is increased confidence in the structure and assessment method.

### Previous analyses

Our earlier studies examined the component structure of the UPDRS Motor Examination section in three separate samples. Two of the samples were composed of patients for whom PD was idiopathic, i.e. not related to or caused by other diseases. One of these samples, PD-On, was examined while patients were on medication (Stebbins & Goetz, 1998) [6] and the other sample, PD-Off, was examined while the patients had been withdrawn from all anti-parkinsonian medications for at least 12 hours (Stebbins, Goetz, Lang, & Cubo, 1999) [7]. We also had at our disposal the motor scores of a sample of patients with a parkinsonian syndrome different from idiopathic PD (progressive supranuclear palsy: PSP).

For each of the samples the component structures were analysed independently, with principal component analyses followed by oblique rotations (Cubo, Stebbins, Golbe, Nieves, et al., 2000) [8]. Reasonably similar sets of components were observed in all three patient groups, but at the same time specific differences were present, which were difficult to evaluate comparatively. In particular, the component structure of the PSP-group deviated from those of the PD groups. It consisted of five components but there were problems in fitting the tremor-at-rest variables; also bradykinesia did not seem as lateralised as in the PD groups.

Detailed comparisons of independently derived components are fraught with difficulties, because there is no common standard with which to compare them. Often approximations to such a common base are used by applying the same rotation technique to the different component structures, but such procedures only work in those cases where there is similarity in structures and their explained variances are high (see, for instance, Bennett, Shannon, Beckett, & Wilson, 1999 [9]). However, when different numbers of components have been extracted, such techniques do not perform adequately. What is necessary is to analyse all samples simultaneously so that differences between the groups can be evaluated with respect to a single solution common to all groups.

### Modelling approach

In the literature several component and factor analysis methods for modelling multiple samples at the same time are available. In the research reported here, the emphasis was on model building using (confirmatory) factor analysis models rather than on exploratory analysis using component models. Because of the knowledge available about the motor section of the UPDRS, explicit model building using factor analysis models was to be preferred here, because it allowed for the formulation and testing of specific models as well as for specifications of detailed restrictions on the parameters and the residuals. In Additional file 1 the differences between exploratory component analysis and confirmatory factor analysis are discussed in more detail including the principles of imposing restrictions for refined model building. Provided distributional assumptions are not grossly violated, this model-building approach can provide detailed insight into the similarities and differences between the three samples. Furthermore, models can be compared in a hierarchical fashion, i.e., starting from a baseline model, all further models can be specified as special cases defined by restrictions on the parameters of the baseline model. Such analyses are especially powerful when considerable substantive information is available from the literature, so that selection and improvement of models can be carried out based on such knowledge in combination with statistical information.

In the present paper, we will show how simultaneous (or multigroup) factor analysis (Jöreskog, 1971) [10] can be used to achieve the desired examination of the similarities and differences between the samples. Of particular interest is the extent to which parts of the simultaneous solution can be restricted, and which parts have to be estimated separately. The search for an adequate and reasonably fitting model is described in some detail, so as to serve as an example for similar endeavours. A more detailed example of this kind can be found in Byrne (2004) [11].

## Methods

### Participants

The UPDRS Motor Examination section items were assessed for three different groups of patients. The three groups consisted of 294 PD patients on medication (PD-On group), 200 PD patients withdrawn from all anti-parkinsonian medications for at least 12 hours (PD-Off group), and 175 patients with Progressive Supranuclear Palsy (PSP-group). The UPDRS data for the series of PD-On patients were collected as part of routine clinical management at a single movement-disorder clinic of a university medical centre. Data for the PD-Off group were collected from patients being evaluated for participation in specific clinical trials at two movement-disorder clinics connected with university medical centres. Data for the PSP-group were collected from three university medical centre based movement disorders clinics (see Stebbins and Goetz (1998) [6], Stebbins et al. (1999) [7], and Cubo et al. (2000) [8] for specifics of the participating sites). Each patient belonged to only one sample. All data from the Motor Examination section of the UPDRS were gathered by trained examiners who met published criteria for inter-rater reliability (Goetz, Stebbins, Chmura, Fahn, Klawans, & Marsden, 1995) [12]. The study was approved by the local Institutional Review Board at Rush University Medical Center, Chicago. Patients were not explicitly asked for their consent, due to the anonymous nature of the data.

### Measures

The Motor Examination section of the UPDRS consists of 27 items, each of which is scored from 0 (normal) to 4 (severe). Note that the scale is defined in such a way that 'normal' persons have zero values on all motor items, so that any deviations from zero indicate impairment of motor functioning. The items are listed in abbreviated form in Table 1, and detailed descriptions can be found in Fahn & Elton (1987) [5], or on the web site of the MDVirtual university [13]. Richards, Marder, Cote, and Mayeux (1994) [14] assessed the inter-rater reliability of the motor section using three neurologists experienced in the administration of the scale. Intraclass correlation coefficients indicated good-to-excellent agreement for speeded repeated movements, resting tremor, arising from a chair, and gait; moderate agreement for action tremor, rigidity, posture, postural stability, and bradykinesia; and poor agreement for speech disorder and facial immobility. Overall their results indicated that satisfactory inter-rater reliability was attainable with the UPDRS motor examination. For the three groups examined in this study, the motor scale had reliabilities between 0.95 and 0.90 using Cronbach's alpha as a measure for internal consistency. Siderowf, McDermott, Kieburtz, Blindauer, Plumb, Shoulson, & Parkinson Study Group (2002) [15] reported a test-retest reliability of 0.90 for the motor examination as measured with the intraclass-coefficient, in accordance with earlier similar assessments.

**Table 1:** Summary statistics of the UPDRS motor items

			*PD-On*				*PD-Off*				*PSP*			
	*UPDRS Motor Items*		***M***	*SD*	*Skew*	*Kurt.*	***M***	*SD*	*Skew*	*Kurt.*	***M***	*SD*	*Skew*	*Kurt.*

1	Speech		**1.40**	0.91	0.37	-.06	**1.72**	0.93	0.33	.03	**2.45**	1.00	0.07	-.92
2	Facial Expression		**1.55**	0.89	0.44	.11	**2.15**	0.85	0.06	-.25	**2.67**	1.02	-0.33	-.58

3	Tremor-at-rest:	H/N	**0.48**	0.73	1.42	1.45	**0.36**	0.76	2.24	*4.71*	**0.02**	0.24	*11.69*	*142.73*
4		RUE	**0.81**	0.93	0.75	-.52	**1.01**	1.19	0.80	-.58	**0.10**	0.42	*4.56*	*22.08*
5		RLE	**0.55**	0.76	1.15	.35	**0.65**	1.01	1.28	.44	**0.03**	0.21	*6.86*	*51.53*
6		LUE	**0.69**	0.87	0.91	-.38	**0.94**	1.19	1.08	.10	**0.10**	0.41	*4.72*	*23.60*
7		LLE	**0.52**	0.73	1.24	.74	**0.65**	1.10	1.60	1.52	**0.03**	0.25	*10.47*	*118.78*
8	Postural tremor	R	**0.66**	0.68	0.87	1.33	**0.93**	0.87	0.69	.02	**0.34**	0.55	1.40	1.02
9		L	**0.64**	0.63	0.46	-.66	**0.98**	0.95	0.69	-.28	**0.34**	0.57	1.65	*2.69*

10	Rigidity	H/N	**1.24**	0.80	0.19	.00	**1.89**	1.07	0.03	-.55	**2.40**	1.19	-0.22	-.91
11		RUE	**1.68**	0.78	-0.24	.16	**1.84**	0.90	-0.21	.07	**1.70**	0.98	0.30	.11
12		RLE	**1.36**	0.80	0.29	.29	**1.58**	1.06	0.01	-.74	**1.34**	1.01	0.35	-.69
13		LUE	**1.63**	0.78	0.26	.54	**1.96**	0.91	-0.25	-.01	**1.61**	1.03	0.23	-.24
14		LLE	**1.39**	0.82	0.44	.60	**1.68**	1.11	0.07	-.76	**1.37**	1.04	0.26	-.83

15	Finger taps	R	**1.58**	0.78	0.34	.55	**2.20**	0.93	0.17	-.46	**1.54**	1.04	0.42	-.37
16		L	**1.59**	0.82	0.69	1.05	**2.38**	1.01	-0.28	-.32	**1.69**	1.12	0.40	-.59
17	Hand movements	R	**1.50**	0.83	0.58	.57	**1.72**	0.94	0.48	-.04	**1.59**	0.98	0.61	-.04
18		L	**1.51**	0.84	0.79	1.06	**1.98**	1.00	0.08	-.49	**1.65**	1.02	0.55	-.22
19	Rapid Alternating. Movements.	R	**1.45**	0.82	0.35	.35	**1.85**	0.97	0.20	-.29	**1.66**	1.06	0.43	-.31
20		L	**1.50**	0.80	0.71	1.14	**2.21**	1.04	-0.16	-.47	**1.76**	1.15	0.53	-.50
21	Leg agility	R	**1.44**	0.83	0.73	.91	**2.05**	1.03	0.02	-.64	**1.57**	1.13	0.42	-.47
22		L	**1.50**	0.85	0.81	1.03	**2.25**	1.03	-0.17	-.55	**1.72**	1.18	0.37	-.59

23	Arise from chair		**1.23**	1.05	1.07	.88	**1.70**	1.45	0.33	-1.28	**2.40**	1.24	-0.11	-1.20
24	Posture		**1.35**	0.86	0.67	.66	**1.75**	1.10	0.26	-.56	**1.47**	1.14	0.31	-.61
25	Gait		**1.28**	0.91	0.66	.29	**2.12**	1.13	0.02	-.71	**2.60**	1.14	-0.42	-.63
26	Postural stability		**0.89**	1.01	1.17	.96	**1.95**	1.23	0.09	-.97	**2.67**	1.12	-0.28	-.92
27	Body bradykinesia		**1.45**	0.97	0.73	.14	**2.46**	1.05	-0.15	-.69	**2.39**	0.98	-0.26	-.92

	Motor Total		**32.9**	14.8	0.38		**44.9**	15.0	0.75		**39.2**	13.9	0.64	

Skew = Skewness; Kurt. = Kurtosis;

Striking values are in *italics*. Standard error skewness approx. 0.15; kurtosis approx 0.32 The items are numbered according to the standard way they appear in the Motor Scale of the UPDRS. R = Right-hand side; L = left-hand side; UE = Upper Extremity; LE = Lower Extremity; H/N = Head and Neck

### Preliminary statistical analyses

Before an analysis of the structure was carried out, for each group numerical characteristics of the distributions of the items of the Motor section were inspected (means, standard deviations, skewness coefficients, and kurtoses). Separate principal component analyses were carried out to investigate the structure per group (following the initial approach taken by Stebbins and co-workers). Details on these analyses can be found in Kroonenberg, Oort, Leurgans, and Stebbins (2000) [16] and in the original studies. A preliminary joint model for the three groups was formulated on the basis of results for the separate component analysis. Clinical knowledge about the origins and manifestation of Parkinson's disease assisted in deciding which types of behaviours should load on the same factor, especially in those cases when the component results were contradictory.

### Simultaneous factor analyses

Because of the differences between Parkinson's and PSP patients, first a model was sought for the two groups of PD patients; only after that had been found was the PSP group was included in the model search for a joint model. The simultaneous factor analyses were carried out with the structural equation modelling program LISREL8 developed by Jöreskog and Sörbom (1996) [17] using the maximum likelihood (ML) estimation method. Additional file 2 contains the LISREL script used to estimate the parameters of the model reported in this paper. Several items in the Motor Examination of the UPDRS were far from normal, with serious skewness and kurtosis (see Table 1). Non-normality poses a problem when choosing a suitable estimation method. We could not use the generally recommended weighted least squares estimation method (Bollen, 1989) [18] because our sample sizes were far too small (around 200). Given violations of the assumption of multivariate normality, the resulting statistic need not have a chi-square distribution, and the standard errors need not be correct. However, the estimates of the model parameters are probably not seriously biased (Bollen, 1989) [18]. A possible alternative to maximum likelihood estimation would have been to use quasi-maximum likelihood estimation with polychoric correlations as the next best alternative for weighted least squares (Satorra, 1992) [19]. However, there has not been much practical experience with the application of this method in multigroup cases.

#### General model search

In the present study, first a relatively unrestricted joint model was formulated for both PD-groups, primarily on the basis of the oblique component solutions. Each item was restricted to load on a single factor. All factors were allowed to be correlated. Furthermore, the residuals similar items whose estimated correlations did not fit adequately, were also allowed to be correlated.

The adequacy of the models was primarily assessed by the Root Mean Squared Error of Approximation (RMSEA; Steiger & Lind, 1980, Browne & Cudeck, 1992) [20,21]. In particular, the RMSEA of the baseline model was taken as a starting point. Only those models whose RMSEA stayed within the confidence bounds of the RMSEA of the baseline model were taken into consideration. In other words, only those restrictions on the parameters of the baseline model were accepted which did not unduly decrease the fit to the data. This procedure is less restrictive than using tests of chi-squared differences to decide whether differences in fit are significant. The main reason for the approach is that we were trying to build an adequate model, rather than testing a number of a priori hypothesized ones. Due to the lack of multivariate normality, it was difficult to use the chi-square distribution to interpret the (non-central) chi-square statistic, and although the RMSEA depends on the normality assumption as well, it is still possible to use the statistic to compare the fit of different models to the same data. Simulation studies (Curran, West, & Finch, 1996) [22] suggested that under non-normality the RMSEA will turn out too high rather than too low. In other words, in these circumstances it tends to be too conservative a measure. Three other fit measures were used as a check on the procedure, in particular the Comparative Fit Index (CFI) and the Tucker-Lewis Index (TLI), and the standardised root mean squared residual, St.RMR (see Hu & Bentler, 1999 [23], for detailed descriptions, references, and information on cut-off values).

#### Model search across samples

To examine similarities and differences between the samples, restrictions were placed on the baseline model. In particular, equality constraints were placed on classes of parameters in the following order: factor loadings, factor correlations, factor variances, and correlations of residuals. By increasing the number of restrictions in an ordered fashion, a set of nested or hierarchical models was defined which could be compared with respect to their fit.

The detailed results of the multigroup factor analysis are reported in its common metric completely standardised solution (for details and considerations with respect to standardising multigroup solutions see Jöreskog & Sörbom, 1996, p. 290 ff [17]). The major characteristic of the common metric standardisation is that the weighted average of the within-group factor covariances is a correlation matrix, unlike the individual factor covariance matrices. This has the advantage that the invariant loading matrices remain invariant in standardised solution. By using the completely standardised solution, the original variables are also standardised to a common metric across groups, which facilitates the comparison of the factor variances and covariances (Jöreskog & Sörbom, 1996, p. 293) [17].

## Results

All three groups showed a substantial burden of motor disability (see Table 1). The mean motor scores were 32.9 (an average of 1.2 points per item) for the PD-On group, 44.9 for PD-Off (an average of 1.7 points per item) and 39.2 for the PSP group (an average of 1.5 points per item). Note that, depending on the state of the disease, patients may show high scores on some but not on all items. The groups all showed substantial differences between individuals: the SD of the total score was around 15 for each group. The distribution of the total motor scores was not skewed for the PD-Off group, but the distributions of the other two groups were mildly positively skewed with a moderate kurtosis. For most items, the PSP-group had the highest skewness, the PD-On groups the lowest, and the PD-off group was intermediate. The five tremor-at-rest items and head/neck tremor did not follow this pattern. Each of the five tremor-at-rest scores had a mean of 0.1 or less in the PSP-group. The SDs for these five items were between 0.21 and 0.42, and skewness and kurtosis coefficients were high. These observations reflected the fact that only 17 of the 175 (10%) PSP patients exhibited any rest tremor. The item-specific SDs were close to 1 for most other items. The elimination of the 17 PSP patients had no great influence on the characteristics of the distributions of the items or total scores, except for the tremor-at-rest and head/neck tremor items. In order to establish whether the high kurtosis for the tremor-at-rest items unfavourably affected the solution, the final model was also examined without these items and the associated tremor-at-rest factor. However, it turned out that the other values and their standard errors were virtually unaffected, so that we concluded that the high kurtosis had no serious influence on the nature of the solution.

### Simultaneous analysis of the PD-On group and PD-Off group

As indicated above, first a common model was sought for the two PD-groups on the grounds that the principal component analyses indicated that there were serious differences between the PD groups and the PSP group. A series of five models was tested; their descriptions and fit statistics are given in Table 2.

**Table 2:** Model selection: Parkinson disease only: PD-On-Group and PD-Off- Group

**Model**	**Description**	χ^2^	***df***	**RMSEA**	**TLI**	**CFI**	**St.RMR**	**Comments**
PD1	Six factors based on component solutions -no constraints across groups; correlated residuals Facial and Speech	1318	592	0.071	.92	.93	0.08	**BASELINE MODEL with 95% confidence interval of RMSEA: 0.065 – 0.077**
PD2	As PD1: Within a factor **all **loadings equal	1578	634	0.078	.90	.91	0.10	Outside interval. PD2; too simple
PD3	As PD1: Loadings PD-On = Loadings PD-Off	1377	613	0.071	.92	.93	0.07	Simpler model than PD2
**PD4**	**As PD3: Also equal factor covariance matrices**	**1450**	**634**	**0.072**	**.92**	**.92**	**0.09**	**Preferred model: Very simple; easy interpretation;**
PD5	As PD4: Also residual variances and residual covariances equal across groups	2497	674	0.100	.82	.83	0.13	Rejected: Oversimplification

Note: RMSEA = Root mean square residual, CFI = comparative fit index, TLI = Tucker-Lewis Index, St.RMR = standardised root mean residual correlation.

#### Baseline model

Initially a model was devised without any correlations between the residuals, i.e. all observed covariances were assumed to be determined by the six factors and their factor correlations. However, due to a very strong correlation between Facial Expression and Speech, no converging model could be found. The only solution was to allow the covariance between the residual factors of these two variables to be estimated as well. Therefore, our *baseline model *was PD1 (see Table 2), which had a chi-square of 1318 with 592 *df *and an RMSEA of 0.071 with a confidence interval of 0.065–0.077; it also had acceptable values for CFI (0.93), TLI (0.92) and a marginally acceptable value for St.RMR (0.08). In this model, the structure of the solution was same for the two groups, but the loadings of each group were unrelated (configural invariance). In order to test whether not only the structure of the model was the same for the two groups but also the values of their parameters, increasingly strict equality constraints were imposed on the parameters across groups. A restricted version of the baseline model was accepted, if its RMSEA stayed within the confidence bounds of the RMSEA of the baseline model. In addition, the GFI, TLI, and the St.RMR were examined in order to evaluate the restricted model with respect to the baseline model.

#### Restricting the baseline model

With Model PD2 an attempt was made to investigate whether a complete equality of all factor loadings on all factors for both groups simultaneously would lead to an acceptable model. Such a model implies an equal weighting of all items in a factor. This restriction gave a gain of 42 *df*. The PD2 model had a chi-square of 1578 with 634 *df *and an RMSEA of 0.078, which was outside the confidence interval of the baseline model. Moreover, both the CFI (0.91) and the TLI (0.90) dropped more than was desirable. As these restrictions turned out to be too severe complete equality was dropped in favour of the requirement of equality of the loadings for both groups. This resulted in a model with a chi-square of 1377 with 613 *df *or a smaller gain of degrees-of-freedom (21 *df*), and a RMSEA of 0.071 (equal to the baseline model) with acceptable values for CFI (0.93), TLI (0.92), and St.RMR (0.07). In other words, this simplified model fitted as well as the baseline model itself, leaving room for further restrictions. The next logical step was to impose equality of factor covariances for both groups, i.e. metric invariance (Model PD4), which provided an acceptable model with a chi-square of 1450 and 634 *df *or a gain of 20 *df*, an RMSEA of 0.072, a CFI of 0.92, a TLI of 0.92 and a somewhat too large St.RMR. Including further restrictions such as equal residual variances and covariances led to unacceptable model fit (Model PD5: chi-square = 2497, *df *= 674; RMSEA = 0.100, CFI = .83, TLI = .82, St.RMR = 0.13).

Thus, Model PD4 was the simplest acceptable model. It had equal loadings for both groups (both the same pattern of factor loadings and equal values for all of them), and also the covariances between the factors are the same. It cannot be further simplified in a systematic and straightforward manner without significant deterioration in fit. It is always possible to include additional parameters on purely empirical grounds but without theory, this seems ill-advised. The only concession to this principle was made for the correlated residual between Facial expression and Speech, because without this correlation an adequate model could not be found at all. In Model PD4 the specific variances of all items were systematically larger in the PD-Off-group than the PD-On-group, suggesting that taking patients off medicine enlarges the specific variability. This can be interpreted to mean that the differences between the patients increase when they are taken off drugs, but that the relationships between the variables were not affected.

#### Conclusion

The equality of factor loadings and their covariances was satisfactory because apparently medication has no serious influence on the structure or relationships between the items of the questionnaire, but only on their means. Thus medication did not change the observed pattern in motor functioning for patients with Parkinson's disease, but only restricted its manifestations.

### Model development: PD groups and PSP-group

The model for the Parkinson's disease groups was taken as the starting point for finding an appropriate model for all three groups together, but this proved to be not feasible. In hindsight this was not surprising, considering the Tremor-at-rest variables. The next candidate was the PD1 Model, which had the same pattern of loadings for all three groups without the requirement of equality for their values (Model PP1, Table 3). Unfortunately, this model was not identified empirically and did not converge, again because of the very small of variability for Tremor-at-rest variables for the PSP-group. This precluded finding proper estimates for them for the PSP-group. By specifying that the factor loadings should be equal across groups, model PP2 did converge with a chi-square of 1949 and 930 *df*, an adequate RMSEA = 0.070 with a 95% confidence interval of 0.065–0.075, a satisfactory CFI = 0.97 and TLI = 0.97, and a largish St.RMR = 0.09. This model served as our baseline model for further exploration. The estimation was only marginally possible because the factor variance for the Tremor-at-rest factor was a tiny fraction above zero (0.01). Due to the lack of Tremor for the PSP-group, equal factor correlation matrices across groups did not produce an acceptable model (Model PP3: chi-square = 2376, *df *= 972, RMSEA = 0.081, falling outside the confidence interval of the baseline model; very low CFI (0.89) and TLI (0.90), and a disastrous St.RMR of 0.22). Equating the correlations for Tremor-at-rest with the other factors for the two PD groups, and estimating those of the PSP-group separately, gave an acceptable solution (Model PP4: chi-square = 2099, *df *= 966, RMSEA = 0.073 – within the confidence interval; CFI = 0.96; TLI = 0.97), although the St.RMR = 0.14 was rather high.

**Table 3:** Model selection: All three groups: PD-On-Group, PD-Off-Group, and PSP Group

		χ^2^	***df***	**RMSEA**	**TLI**	**CFI**	**St.RMR**	**Comments**
PP1	Same model as PD1	--	--	--	--	--	--	Empirically not identified
PP2	As PP1: Loadings PD-On = Loadings PD-Off = Loadings PSP	1949	930	0.070	0.97	0.97	0.09	**Baseline model 95% Confidence Interval of RMSEA: 0.065 – 0.075**
PP3	As PP2: Also factor correlation matrices equal^1^	2376	972	0.081	0.89	0.90	0.22	Outside confidence limits PP2
**PP4**	**As PP3: In PSP all factor (co)variances related to the factor Tremor estimated rather than set equal to those of the PD groups**	**2099**	**966**	**0.073**	**0.96**	**0.97**	**0.14**	**Within confidence limits of PP2 Preferred model**

^1^The model modification indices of the factor variance for Tremor-at-rest were moderately large to large (PSP: 70; PD-On: 19; PD-Off : 12) indicating that the common values were not very accurate for these variances, suggesting separate estimations of these quantities. RMSEA = Root mean square residual, CFI = comparative fit index, TLI = Tucker-Lewis Index, St.RMR = standardised root mean residual correlation.

The equality of the factor loadings for the PD-groups and the PSP-group was in itself remarkable. It suggests that for the few (17) PSP patients who did show tremor-at-rest, the relationships between the variables making up this factor were not different from those found for the patients with Parkinson's disease. However, it has to be remarked that given the small number of subjects there was not much power to find differences.

### Conclusion of the model search

It was possible to find a very simple, easily interpretable model that specified equality of factor loadings and factor (co)variances across groups, except for the (co)variances of Tremor-at-rest variables, which needed separate estimation for the Parkinson's and PSP patients. Some complexity still remained, especially due to the residual variances, but they are not the most important part of the model.

### Model fit

The final overall model for all three groups together is presented in Table 4. It has a chi-square of 2099 with 966 *df*. The Root Mean Square Error of Approximation (RMSEA) was 0.073, which is generally considered a reasonable fit (see Browne and Cudeck, 1992; Hu and Bentler, 1999) [21,23]. Both the CFI (0.96) and the TLI (0.97) had acceptable values; only the St.RMR was rather high with a value of 0.14. The contributions of the PD-On, PD-Off, and PSP groups to the overall chi-square were 797, 681 and 621, or 2.7, 3.4 and 3.5 per subject respectively, showing that the fit was best for the PD-On-group, and more or less equal for the PD-Off and the PSP-group. However, these differences in the lack of fit were not large and the model fitted reasonably both for all groups separately and overall. This means that we were able use the parameters to describe the differences and similarities between the groups within a unified framework.

**Table 4:** Simultaneous analysis: Final model (Common metric completely standardised solution)

			1	2	3	4	5	6	Residual variances		
			
			Axial Dysfunctioning	Tremor-at-rest	Postural tremor	Rigidity	Brady-kinesia Right	Brady-kinesia Left	On	Off	PSP
3	Tremor-at-rest:	H/N		0.78					0.39	0.63	0.13
4		R Upper		0.69					0.50	0.85	0.19
6	*Extremities:*	L Upper		0.74					0.33	0.88	0.18
5		R Lower		0.70					0.37	1.09	0.06
7		L Lower		0.73					0.21	1.17	0.08
8	Postural tremor	R			0.90				0.13	0.42	0.04
9		L			0.75				0.27	0.81	0.31
											
10	Rigidity	Head/Neck				0.67			0.22	0.65	**1.02**
11		R Upper				0.68			0.40	0.62	0.69
12	*Extremities:*	L Upper				0.76			0.24	0.64	0.49
13		R Lower				0.70			0.38	0.59	0.66
14		L Lower				0.77			0.20	0.64	0.50
											
15	Finger taps	R					0.81		0.21	0.44	0.45
16		L						0.82	0.22	0.39	0.47
17	Hand movements	R					0.90		0.14	0.29	0.18
18		L						0.90	0.15	0.27	0.18
19	Rapid alt. mov.	R					0.82		0.18	0.49	0.40
20		L						0.83	0.15	0.45	0.40
21	Leg agility	R					0.72		0.22	0.67	0.71
22		L						0.68	0.31	0.64	0.79
											
23	Arise from chair		0.82						0.22	0.45	0.34
24	Posture		0.72						0.20	0.42	**1.04**
25	Gait		0.82						0.15	0.38	0.60
26	Postural stability		0.77						0.28	0.49	0.49
27	Body bradykinesia		0.79						0.34	0.40	0.45
1	Speech		0.60						0.50	0.69	0.80
2	Facial expression		0.60						0.53	056	0.92

The order of some items has been rearranged from their order in the UPDRS to show some patterns more clearly. R = Right-hand side; L = left-hand side; H/N = Head and Neck. All *t*-values for the loadings were greater than 12.9; for the residual variances they were typically around 8. The smallest *t*-values were for Right-hand side Postural Tremor (PD-On: 2.2; PD-Off: 4.2; PSP: 0.52); these three residual variances were the only ones with *t*-values below 4.5.

### Model characteristics

**Figure 1:**
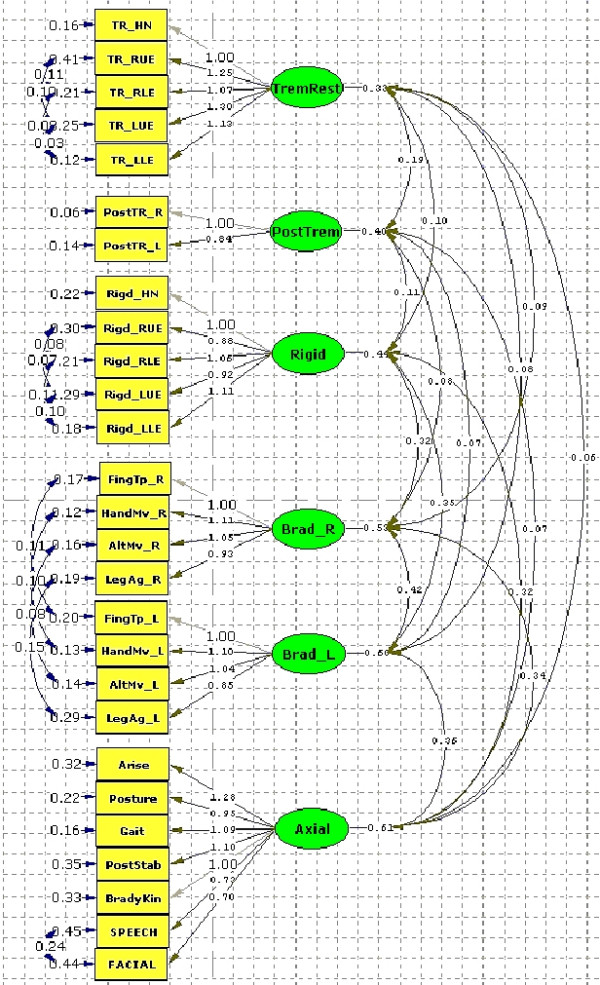
**Complete factor model for the simultaneous modelfor all three groups. **The values are those from the common metric completely standardised solution. Factor loadings and factor correlations are common to all groups except the factor correlations of the Tremor-at-Rest factor, which are those of the Parkinson's disease groups. The residual variances and covariances are group-specific and the ones shown are those of the PD-On group.

The overall model – Model PP4 (Figure 1) had the following characteristics: (1) The items have the same factor loadings for all groups, both with respect to pattern and with respect to the actual values, and all of them were significant; (2) except for the Tremor-at-rest factor, all factor variances and covariances had the same values for all groups; moreover, all (co)variances were significant, except the (co)variances with Tremor-at-rest for the PSP-group; (3) the factor variances and covariances for Tremor-at-rest had the same values for the PD-groups, but those for the PSP-groups had to be estimated separately; (4) the residual variances specific to each item (i.e. independent of the common variance) in each group had to be estimated separately; (5) the correlations between specified residuals had separate estimates for each group (see also Method section). In other words, the same model (common factors and factor correlations) fitted all three groups reasonably well except for the tremor-at-rest variables.

### Residual variances

The residual variances of the items provided information on the extent to which the fitted model succeeded in reproducing the variances of the items. As all residual variances for the PD-Off-group were larger than those for the PD-On-group, the conclusion must be that the former group had much more unexplained variance, which is consistent with the idea that different patients react in different ways when off medication. The residual variances for the PSP-group, except for Tremor-at-rest, were generally closer to the PD-Off-group than to those of the PD-On-group (Table 4) suggesting that the behaviour of PSP-patients is rather more variable in a non-systematic way. For the PSP-group large residual variances for Posture and for Head & Neck Rigidity indicated that much variability in these variables could not be captured by the common factors.

### Factor correlations

The factor correlation matrix (Table 5) has a simple interpretation. Six correlations above 0.5 belonged to a block of four *Non-Tremor *factors: Axial Dysfunctioning, Rigidity and Left & Right Bradykinesia. The two remaining factors were the two *Tremor *factors: Tremor-at-rest and Postural Tremor. In both of the PD groups, the Tremor factors were highly correlated (0.52), but not surprisingly, given the fact that the correlations were based on very few patients, the correlation of the Tremor factors in the PSP-group was low (0.13). The highest cross-correlation between the Tremor and Non-Tremor factors was also low (0.26), indicating that the motor section of the UPDRS primarily consists of two reasonably independent parts, tremor and bradykinesia.

**Table 5:** Simultaneous analysis: Factor variances and correlations

		2 (PSP)	2 (PD)	3	1	4	5	6
		
		Tremor-at-rest	Tremor-at-rest	Postural Tremor	Axial Dysfunctioning	Rigidity	Brady-kinesia Right	Brady-kinesia Left
2	Tremor-at-rest	(0.01) †	(0.33)					
3	Postural Tremor	0.04††	**0.61**	(0.40)				
								
1	Axial Dysfunctioning	-0.03††	0.13	0.14	(0.61)			
4	Rigidity	0.01††	0.29	0.26	**0.61**	(0.44)		
5	Bradykinesia Right	0.00††	0.24	0.18	**0.60**	**0.67**	(0.53)	
6	Bradykinesia Left	0.00††	0.22	0.14	**0.60**	**0.67**	**0.74**	(0.60)

Factors have been ordered to emphasize the block pattern in the correlations.

Variances of factors are on the diagonal between brackets and the correlations among factors are in the off-diagonal cells. † indicates *t*-value <3, †† *t*-value <2

### Residual covariances

Residual covariances measure what two variables have in common that is not captured by the common factors (Table 6). The covariances between left and right leg agility (0.16 for PD-On, 0.42 for PD-Off, and 0.57 for PSP) indicated that there was less lateralisation in leg-agility than was suggested by the two left and right bradykinesia factors, which themselves were correlated (0.74). This was especially true for the PSP-group (0.57).

**Table 6:** Residual covariances: Common metric completely standardised solution

Residual correlation between	Finger taps			Hand movements			Rapid alternating movements			Leg Agility		
				
	On	Off	PSP	On	Off	PSP	On	Off	PSP	On	Off	PSP
Right and left-hand side	0.13	0.20	0.30	0.11	0.13	0.11	0.09	0.24	0.29	0.16	0.42	0.57

Numbers indicate the residual correlations. For instance, the first number 0.13 indicates the correlation between the residuals of Finger tapping between the right-hand and the left hand for the PD-On-group. All *t*-values are above 3.

**Figure 2:**
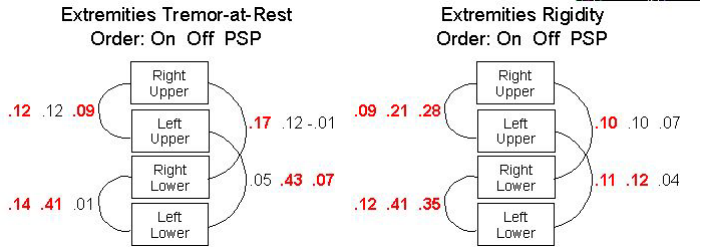
**Residual covariances for the Tremor-at-Rest variables and the Rigidity variables**. Values are those from the common metric completely standardised solution. The bold values have *t*-values larger than 3. The order of the three numbers is always: PD-On, PD-Off, PSP.

Except for Tremor-at-rest and hand movements, the residual covariances between the same left and right activities for the PSP-group were generally higher than in the other two groups, indicating less lateralization of the disease in that group compared to the Parkinson's groups (Figure 2). Note that this was particularly true for leg agility. In addition, the residual covariances were somewhat higher for the PD-Off-group than for the PD-On-group, probably indicating that the drugs also increased some of the lateralization of the disease in Parkinson's patients. Finally, as mentioned earlier, speech and facial expression were more strongly correlated than could be captured by the single Axial Dysfunctioning factor, and the residual correlations for the PD-On, PD-Off and PSO groups were more or less equal (0.28, 0.23, and 0.29, respectively).

## Discussion

In this paper, three different groups of patients, patients with Parkinson's disease on medication (PD-On), patients with Parkinson's disease withdrawn from anti-parkinsonian medication for at least 12 hours (PD-Off), and patients diagnosed with a specific Parkinsonism syndrome: Progressive Supranuclear Palsy (PSP), were evaluated by means of the Motor Examination section of the Unified Parkinson's Disease Rating Scale – UPDRS (Fahn & Elton, 1987) [5]. The factor structure of this scale was examined with simultaneous factor analysis for several groups. This approach extended the work of Stebbins and co-workers (Stebbins and Goetz, 1998; Stebbins, et al., 1999; Cubo et al., 2000) [6-8], who presented the oblimin transformed component solutions for each of the three groups separately.

The central result was the finding that the relationships between the variables in all groups were the same, i.e. the same factor loadings matrix fitted all three groups. Not only the patterns but also their values for all of the groups were the same. In addition, the covariances between the factors were the same for each group. The only exceptions were the (co)variances for tremor-at-rest variables, which were different for the PSP-group due to extremely infrequent positive scores for Tremor-at-rest. There was also evidence for less lateralization of the symptoms in the PSP-group. The major differences between the groups of patients were located in the means on the items, in the tremor-at-rest (co)variances, and in the degree of lateralization. Moreover, the scores of the PD-Off-group were more variable than those of the PD-On-group, which was most likely related to medication.

One particular problem that could be properly addressed by the simultaneous analysis was the question of side-to-side variation in bradykinesia. In their separate analyses, the two PD groups had separate components for the two sides, whereas only a single component was present in the analysis of the PSP-group, as side-to-side variation is much less prominent in PSP-patients. In the multigroup solution separate factors were modelled for the left and right bradykinesia, which were allowed to be correlated.

### Comparison with other studies

At least two other studies explicitly reported principal component analyses of the UPDRS (Bennett, Shannon, Beckett, & Wilson, 1999; Louis, Tang, & Mayeux, 2004 [9,24]). The former authors presented the component spaces of three analyses of different general population samples of elderly persons. In general, the items grouped as Tremor, Axial Dysfunctioning, Rigidity, and Bradykinesia, with different analyses showing a splitting of some components especially the Tremor one. A slight problem with the analyses was that some reported components only represented two items, thus essentially representing the correlation between the two items. The latter study reported the principal component results on a subset of the Motor Section with the components Rigidity and Axial Dysfunctioning and a third component representing the single Tremor-at-rest item. Given that these studies did not perform confirmatory analyses, more detailed comparisons are hard to make.

During the review process an unpublished thesis (Štochl, 2005) [25] came to our attention, in which confirmatory factor analysis was applied to two groups of patients with Parkinson's disease similar to our samples, i.e. one on medication and one off medication. Štochl reported a simultaneous analysis with factors Facial (Speech and Facial Expression), Tremor (both At rest and Postural), Rigidity, Bradykinesia, Axial dysfunctioning as well as separate Left and Right factors. Fitting his model to our data led to not quite acceptable models (RMSEA = .09 and RMSEA = .10) when fitted to the groups separately. A simultaneous analysis of both PD groups did not converge. A full-scale comparison of the two models would, however, take us outside the scope of the present paper. The availability of the Czech data might enable cross-nation comparisons, provided it can be established that scoring practice across countries are the same.

## Conclusion

### Substantive conclusions

The results indicated a stable factor structure for the UPDRS Motor Examination Section. Across the three samples, consistent domains of motor function assessment were obtained while maintaining clinical differentiations between the groups. Thus the PSP results indicated rare tremor-at-rest and decreased laterality of most motor signs. Both of these findings are consistent with the clinical manifestations of PSP. The general separation of tremor-related features from non-tremor related features supports the differentiation of two cardinal signs of all movement disorders: tremor and bradykinesia.

Our findings of a stable factor structure and consistent factor loadings across medicated and non-medicated PD patients as well as patients with PSP, indicate that the UPDRS Motor Examination Section provides a valid assessment of motor function independent of specific disease status. The similarity of the UPDRS factor structures in a large sample of older persons without movement disorders (Bennett et al, 1999) implies a clinical utility for the scale beyond its original intent of assessing motor function in patients with Parkinson's disease. However, a further detailed confirmatory study is necessary to describe numerically to which extent the structures are similar and to which extent they are different. In addition, reports of specific UPDRS domain associations with increased risks for other age-related diseases (e.g., Alzheimer's disease, see Bennett et al, 1999 [9]; Louis et al., 2004 [24]) demonstrates the potential use of this scale to assess early markers of adverse consequences to motor impairments. Again detailed confirmatory analyses are needed to make this statement more precise.

### Methodological conclusions

Even though we have concluded the model search with an, in our opinion, acceptable common model for all groups, some doubt remains because none of the baseline models exhibited good fit with an RMSEA below 0.05, but only acceptable fit with an RMSEA just above 0.70. However, Browne and Cudeck [21] considered that an RMSEA below 0.08 is still satisfactory. It also became clear that the approach is sensitive enough to reject models in which the restrictions went too far.

Another concern of the present analysis is the maximum likelihood estimation procedure in the light of the evident kurtosis in some of the variables. However, a re-analysis with the elimination of the most deviating variables showed extreme stability both in the estimates and the standard errors of the parameters. In recent years other estimation procedures have been suggested for confirmatory factor analyses with small numbers of subjects, such as quasi-maximum likelihood in combination with polychoric correlations (Satorra, 1992) [19]. However, the more complex situation of the multigroup analysis combined with the requirement of analysing covariance matrices rather than correlation matrices, makes it uncertain how such a procedure behaves in the present situation. Clearly, once such procedures have been established, it would be extremely useful to use them in multigroup analyses as presented here.

The major advantage of analyzing the data of the three groups jointly using simultaneous (or multigroup) factor analysis is that by treating them within a single analysis, we have a base for comparison. Separate component analyses are difficult to compare numerically, for instance because of the differing number of components. Moreover, the simultaneous factor analyses allowed the testing of equality of factor structure, correlations between factors, and possible equality of residual variances. However, such a model search is not without its difficulties, as was for instance shown by the obligatory inclusion of a residual covariance between two variables. Moreover, in several attempted models no convergent solution was found and other solutions had to be constructed to fit the model to the data. Some early variants of the models that were suggested by the methodologists could find no favour in the eyes of the medical researchers, and they came up with different suggestions that worked better than those of the methodologists. This emphasizes that without substantive input structural equation modelling can be a hazardous business.

Finally, ideally the models that emerged here should be tested further on independent samples. However, already for the present samples, data had to be combined from several clinics to make up the numbers. With a larger number of clinics and subjects, ascertaining of differential effects of variance due to rater and/or clinic could be assessed. However, considerable effort and money will be necessary to collect samples of sizes similar to ours. Actually, larger samples would be preferable as the present samples were already on the small side for comfortable structural equation modelling. However, the equality restrictions ensured that for the main parameters the estimation was based on all 669 patients.

## Competing interests

The author(s) declare that they have no competing interests.

## Authors' contributions

CCG, EC, and GTS conceived of the study, participated in its design, coordination and the data collection in all its aspects. PMK and SEL drafted the manuscript. PMK and FJO performed the statistical analyses. All authors read and approved the final manuscript.

## Pre-publication history

The pre-publication history for this paper can be accessed here:



## Supplementary Material

Additional file 1 Principal component analysis and confirmatory factor analysis and the use of restrictions: A brief comment. In this file the differences between exploratory component analysis and confirmatory factor analysis are discussed in more detail than in the paper and the principles of imposing restrictions for refined model building are explained. Click here for file

Additional file 2 Simplified LISREL script for final analysis. The file contains a simplified version of the LISREL script which was used to estimate the parameters of the model reported in this paper. Full detailed scripts for all analyses can be obtained from the first author. Click here for file
