# Supplementary material for: Motor function in Parkinson's disease and supranuclear palsy: simultaneous factor analysis of a clinical scale in several populations
Source: BMC Med Res Methodol. 2006 Jun 13;6:26. doi: 10.1186/1471-2288-6-26 (PMC1538612; doi:10.1186/1471-2288-6-26)
Supplement: Additional file 1 — Principal component analysis and confirmatory factor analysis and the use of restrictions: A brief comment. In this file the differences between exploratory component analysis and confirmatory factor analysis are discussed in more detail than in the paper and the principles of imposing restrictions for refined model building are explained. [file 1471-2288-6-26-S1.doc]

### Additional file 1: Principal component analysis and confirmatory factor analysis and the use of restrictions: A brief comment

Most previous analyses of the Motor Section of the UPDRS have been performed using principal component analysis followed by some kind of rotation of the component loadings, mostly varimax. Unfortunately, such an analysis is often referred to as factor analysis, which in its purest form is a different technique. Principal component analysis is an exploratory technique used to search for a parsimonious representation of the set of variables. Geometrically, this means that the variables, which span a high-dimensional space, are depicted in or projected into a low-dimensional space. Once found, the low-dimensional space is searched for meaningful groups of variables via either orthogonal or oblique rotations. The resulting rotated axes are often, sometimes inappropriately, referred to as factors. In comparing solutions from different samples, the difficulty is exactly the exploratory character of the solutions. Because there is no common standard or specific model to compare the analyses with, numerical evaluation of the solutions with respect to each other is very difficult, certainly without access to the original data. Therefore, most studies using principal component analysis show such comparisons in a qualitative, descriptive way.

Confirmatory factor analysis starts at the other end. The researcher formulates hypotheses for theoretical constructs such as Bradykinesia, Axial Dysfunctioning, etc. and devises observable variables (such as Finger tapping or Gait and Posture) to measure these constructs, but as such measurements only do this imperfectly there are measurement errors. The system of observed variables and their relationships with the hypothesised constructs is called the measurement model. Given this measurement model and the relationships between the theoretical constructs, or the structural model, the researcher attempts to verify the acceptability of the entire model by confronting the implication of the model, i.e. the values of the data if the model were true, with the real data themselves. Because theoretical constructs are usually correlated, because some variables are influenced by several factors, and because due to the similarity of measurement instruments variables can be more correlated than can be explained by the factors, complicated models have to be built to represent the complete system of latent variables (factors), observed variables, measurement errors, and their correlations, as is evident from Figure 1. Because of this complexity, it is difficult to find a well-fitting model, and models have to be finely tuned to make the reconstructed data on the basis of the model correspond well to the real ones. The main part of the paper deals with this fine tuning of an otherwise fairly obvious basic model.

As the models are designed to fit the covariance matrices of several groups, it is possible to formulate hypotheses about the equality of their factor structures. One may test whether the factor loadings have the same pattern across groups. In other words, the same variables load on the same factors but the loadings need not have the same values in each group (*configural invariance*). One can restrict the solutions further by demanding that the actual values of the loadings are also the same (*partial metric invariance*). In such a case the factors determine the variables in exactly the same manner in all samples. When not only the factor loadings but all parameters in the model are the same in all samples, one has *complete metric invariance*. The advantages of placing restrictions is that (1) equality hypotheses can be tested; (2) the number of parameters in the model is reduced to allow for more acurate estimation because there are more degrees of freedom; (3) the standard errors for the common parameters are smaller because they are based on more data; (4) the interpretation for models with less parameters is simpler.

That the present model is not the only one conceivable, is demonstrated by the work of Štochl who presented a different model, which has similarities but also substantial differences with the basic structure found here. Which of the models will hold up better in other samples will have to be investigated in depth and will require the original data, or at least the complete covariance matrices. In contrast with exploratory component analysis, in confirmatory factor analysis it is the model that is the standard for comparison between studies; it can be numerically evaluated against other samples. Our brief evaluation of the application of Štochl's model to our data is a case in point.
