# Supplementary material for: Motor function in Parkinson's disease and supranuclear palsy: simultaneous factor analysis of a clinical scale in several populations
Source: BMC Med Res Methodol. 2006 Jun 13;6:26. doi: 10.1186/1471-2288-6-26 (PMC1538612; doi:10.1186/1471-2288-6-26)
Supplement: Additional file 2 — Simplified LISREL script for final analysis. The file contains a simplified version of the LISREL script which was used to estimate the parameters of the model reported in this paper. Full detailed scripts for all analyses can be obtained from the first author. [file 1471-2288-6-26-S2.doc]

### Additional file 2: Simplified LISREL script for final analysis

| **Group 1:** Parkinson-On: 6 factors - BR-L and BR-R | *Title for Group PD-On* |
| --- | --- |
| DA NI=27 NO=294 MA=cm NG=3 | *Data specification: 27 variables, 294 patients; covariance matrix; three groups* |
| CM File=On.CM | *Input file name for covariances* |
| LA  Speech Facial  RTHN RTRUE RTRLE RTLUE RTLLE  ATR ATL  RigHN RigRUE RigRLE RigLUE RigLLE  FinTapR FinTapL  HandR HandL,AltMovR AltMovL  LegAgilR LegAgilL Arise  Posture Gait PostStab Brady / | *Labels for 27 UPDRS items* |
| MO NX=27 NK=6 LX=fu PH=sy,fr TD=sy,fr | *Model: 27 variables, 6 factors, full pattern matrix (LX), symmetric factor covariance matrix (PH), symmetric residual covariance matrix (TD) [fr =free= parameters will be estimated]* |
| LK  Axial Rest AT Rigid Brady-R Brady-L | *Labels for factors* |
| PA LX  1 0 0 0 0 0  1 0 0 0 0 0  0 0 0 0 0 0 ! LX(3,2) fixed at 1  0 1 0 0 0 0  0 1 0 0 0 0  0 1 0 0 0 0  0 1 0 0 0 0  0 0 0 0 0 0 ! LX(8,3) fixed at 1  0 0 1 0 0 0  0 0 0 0 0 0 ! LX(10,4) fixed at 1  0 0 0 1 0 0  0 0 0 1 0 0  0 0 0 1 0 0  0 0 0 1 0 0  0 0 0 0 0 0 ! LX(15,5) fixed at 1  0 0 0 0 0 0 ! LX(16,6) fixed at 1  0 0 0 0 1 0  0 0 0 0 0 1  0 0 0 0 1 0  0 0 0 0 0 1  0 0 0 0 1 0  0 0 0 0 0 1  1 0 0 0 0 0  1 0 0 0 0 0  1 0 0 0 0 0  1 0 0 0 0 0  0 0 0 0 0 0 ! LX(27,1) fixed at 1 | *Specification of parameters to be estimated in pattern matrix (LX): 1 is estimated; 0 is not-estimated or fixed. LX has 27 rows (items) and 6 columns (factors).*  *All fixed values are set (implicitly) equal to 0, accept the value of one of the items for each factor which is set equal to 1. Such an item thus has a coefficient of 1 on the factor and fixes the scale of that factor (see next section)* |
| VA 1 LX(1,1) LX(2,1) LX(23,1)-LX(27,1)  VA 1 LX(3,2)-LX(7,2)  VA 1 LX(8,3)-LX(9,3)  VA 1 LX(10,4)-LX(14,4)  VA 1 LX(15,5) LX(17,5) LX(19,5) LX (21,5)  VA 1 LX(16,6) LX(18,6) LX(20,6) LX (22,6) | *Starting values for pattern matrix LX. All estimates start at 1. Also one fixed value per factor is set at 1.* |
| PA TD  1  1 1  0 0 1  0 0 0 1  0 0 0 1 1  0 0 0 1 0 1  0 0 0 0 1 1 1  0 0 0 0 0 0 0 1  0 0 0 0 0 0 0 0 1  0 0 0 0 0 0 0 0 0 1  0 0 0 0 0 0 0 0 0 0 1  0 0 0 0 0 0 0 0 0 0 1 1  0 0 0 0 0 0 0 0 0 0 1 0 1  0 0 0 0 0 0 0 0 0 0 0 1 1 1  0 0 0 0 0 0 0 0 0 0 0 0 0 0 1  0 0 0 0 0 0 0 0 0 0 0 0 0 0 1 1  0 0 0 0 0 0 0 0 0 0 0 0 0 0 0 0 1  0 0 0 0 0 0 0 0 0 0 0 0 0 0 0 0 1 1  0 0 0 0 0 0 0 0 0 0 0 0 0 0 0 0 0 0 1  0 0 0 0 0 0 0 0 0 0 0 0 0 0 0 0 0 0 1 1  0 0 0 0 0 0 0 0 0 0 0 0 0 0 0 0 0 0 0 0 1  0 0 0 0 0 0 0 0 0 0 0 0 0 0 0 0 0 0 0 0 1 1  0 0 0 0 0 0 0 0 0 0 0 0 0 0 0 0 0 0 0 0 0 0 1  0 0 0 0 0 0 0 0 0 0 0 0 0 0 0 0 0 0 0 0 0 0 0 1  0 0 0 0 0 0 0 0 0 0 0 0 0 0 0 0 0 0 0 0 0 0 0 0 1  0 0 0 0 0 0 0 0 0 0 0 0 0 0 0 0 0 0 0 0 0 0 0 0 0 1  0 0 0 0 0 0 0 0 0 0 0 0 0 0 0 0 0 0 0 0 0 0 0 0 0 0 1 | *Specification of parameters to be estimated in residual covariance matrix (TD): Diagonal elements variances. Off-diagonal ones indicate that residual covariances are included, e.g. between left and right leg agility.*  *TD is a 27 items by 27 items lower triangular matrix. Diagonal values are the residual variances and the 1s indicate the residual covariances to be estimated.*  *In our analyses we had to manipulate the starting values of the residual covariance matrix in order to start close enough to the final solutions, so as not to get improper solutions. (Details upon request.)* |
| OU | *Output* |
| **Group 2**: Parkinson-On: 6 factors - BR-L and BR-R  DA NI=27 NO=200 MA=cm  CM File=Off.CM  MO NX=27 NK=6 LX=in PH=in TD=ps  OU | *Specifications for PD-Off Group*  *Pattern matrix = invariant; Factor covariances = ­invariant; Residual covariances (TD)=pattern same, starting values same.* |
| **Group 3**: PSP: 6 factors - BR-L and BR-R  DA NI=27 NO=175 MA=cm  CM File=PSP.CM  MO NX=27 NK=6 LX=in PX=in TD=ps | *Specification for PSP-Group (as above)* |
| FR ph 2 1 ph 2 2 ph 3 2 ph 4 2 ph 5 2 ph 6 2 | *Factor covariances of the second factor (Tremor-at-rest) with the other factors are made "free", i.e. not equal to those of the other groups, and estimated separately. Other elements are equal to those of the PD groups.* |
